# Supplementary material for: Single-molecule kinetic locking allows fluorescence-free quantification of protein/nucleic-acid binding
Source: Commun Biol. 2021 Sep 15;4:1083. doi: 10.1038/s42003-021-02606-z (PMC8443601; doi:10.1038/s42003-021-02606-z)
Supplement: Supplementary file 2 — Supplementary Information [file 42003_2021_2606_MOESM2_ESM.pdf]

Supplementary information

Supplementary Figures S1-S7
Supplementary Tables S1-S2
Supplementary Notes S1-S3

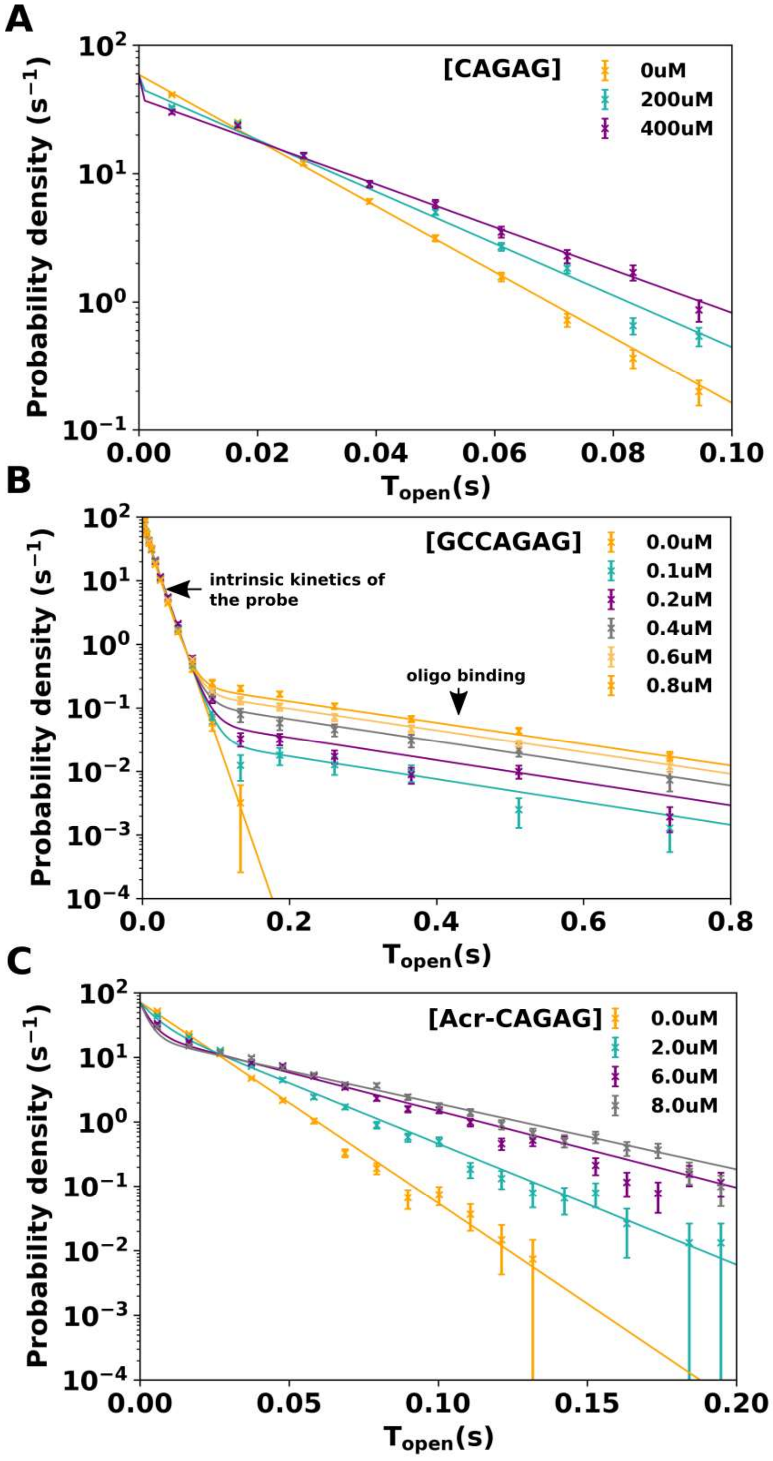

Figure S1. **Distribution of the times spent by the DNA probe in its open state as a function of the concentration of oligonucleotides.** For all oligonucleotides, a unique fit to the expected distribution (Supplementary Note S1) is performed. All concentrations are fitted together. **A**. 5-mer CAGAG. The kinetics of hybridization of the oligonucleotide being much faster than the kinetics of the probe, all distributions follow single exponential laws. k_{on} and k_{off} cannot be inferred. (Supplementary Note S1, limiting case 2). **B**. 7-mer GCCAGAG. The kinetics of hybridization of the oligonucleotide being much slower than the kinetics of the probe, all distributions follow double exponential laws. k_{on} and k_{off} can be inferred. (Supplementary Note S1, limiting case 1). **C**. 5-mer CAGAG with 5' acridine modification. Intermediate case. k_{on} and k_{off} can be inferred but with a smaller precision due to the small proportion of short times.

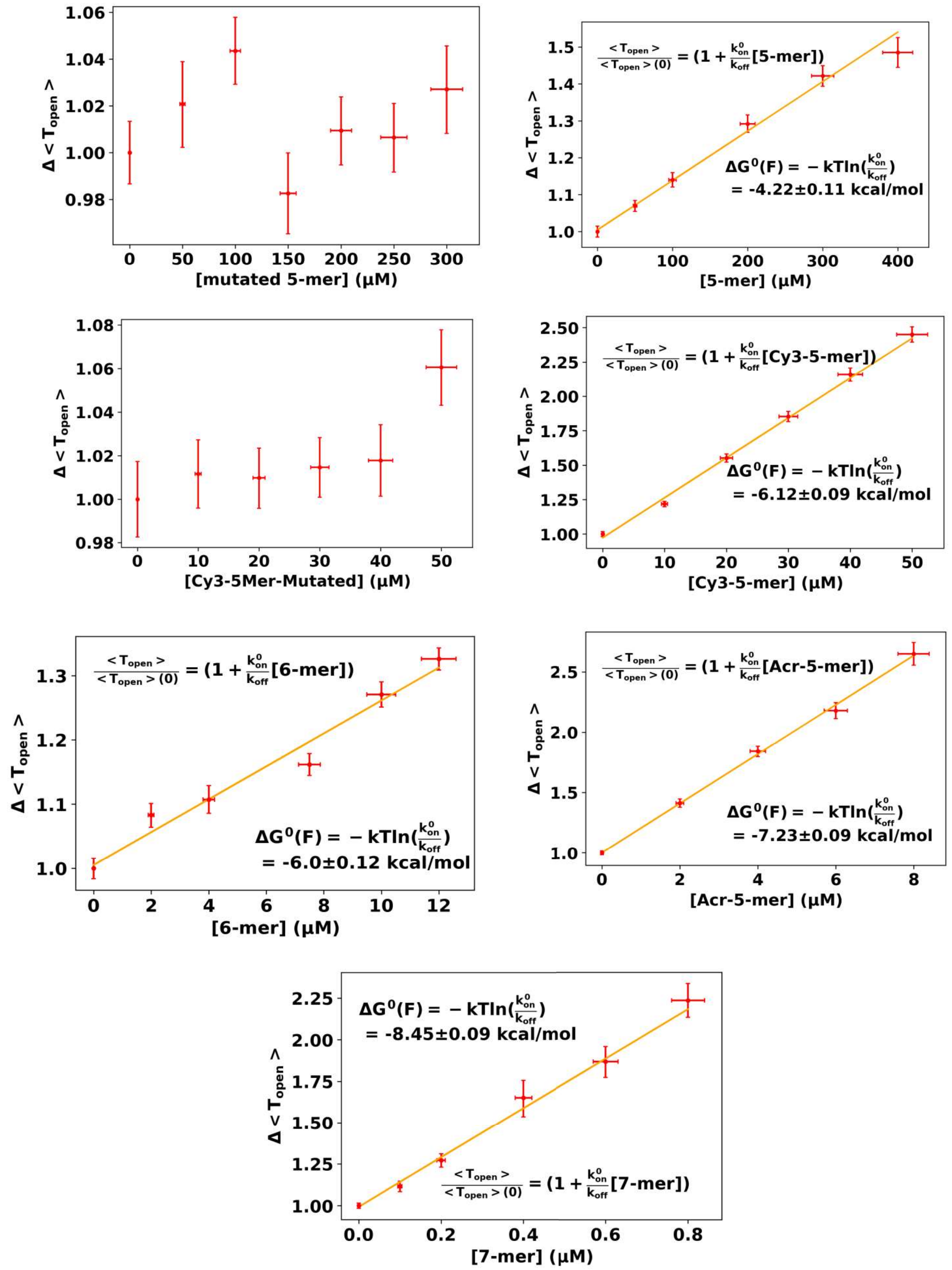

Figure S2. Average times spent by the fluctuating probe in its open state $\overline{T_{\text{open}}}$ as a function of oligonucleotide nature and concentration. Error bars are computed by bootstrap resampling. Errors on the fit are computed using the covariance matrix. Averages are performed on samples containing at least 4000 independent times. Mutated 5-mers (CACAG) have a mismatch at the third position compared to the oligonucleotides (CAGAG) that are complementary to the hairpin sequence.

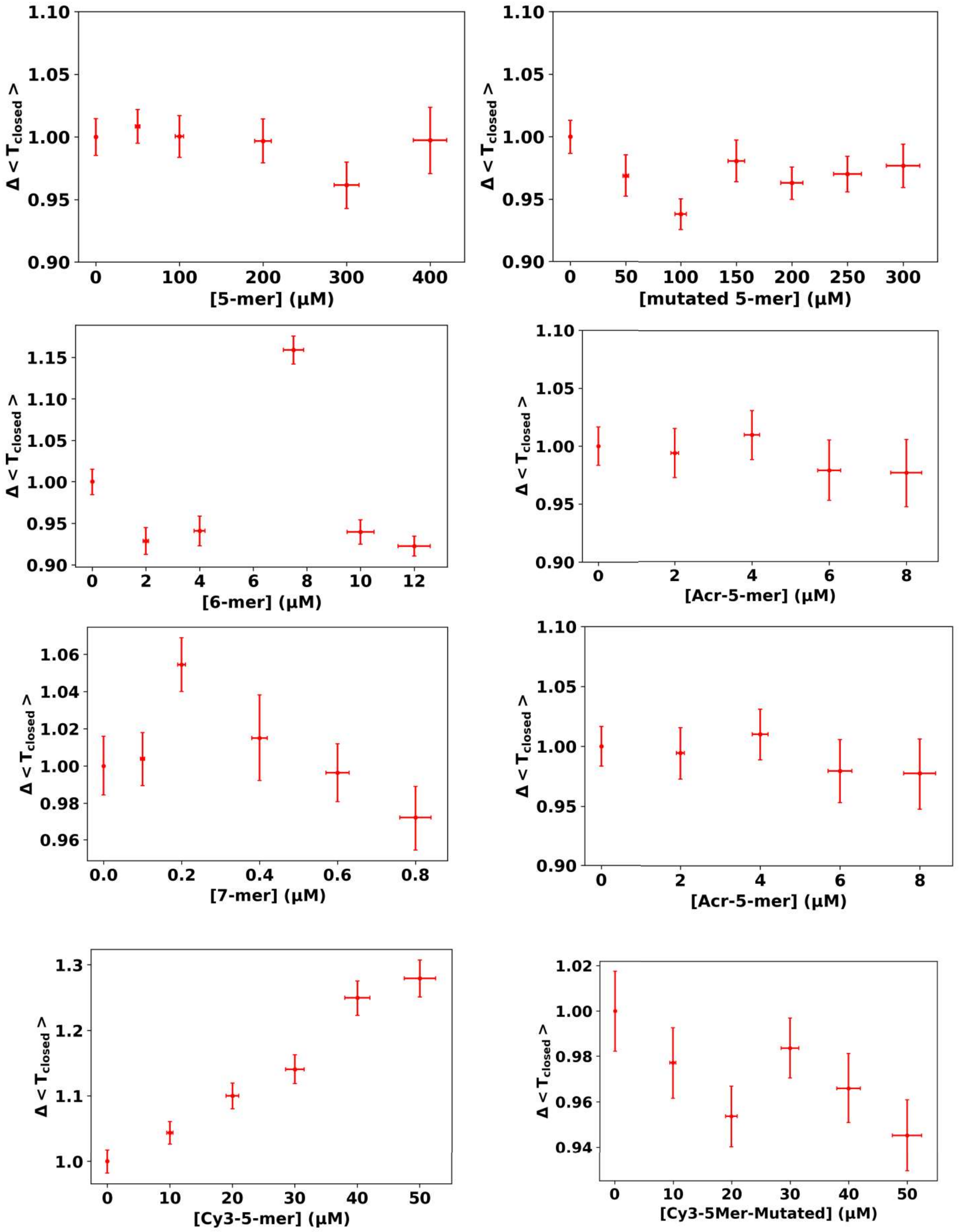

Figure S3. Dependence of the mean times spent by the fluctuating probe in its closed state as a function of oligonucleotide concentration. No change can be observed, with the notable exception of the 5-mer with 5’ Cy3 modification, that stabilizes the closed duplex state. Error bars are computed by bootstrap resampling. Averages are performed on samples containing at least 4000 independent times. Mutated 5-mers (CACAG) have a mismatch at the third position compared to the oligonucleotides (CAGAG) that are complementary to the hairpin sequence.

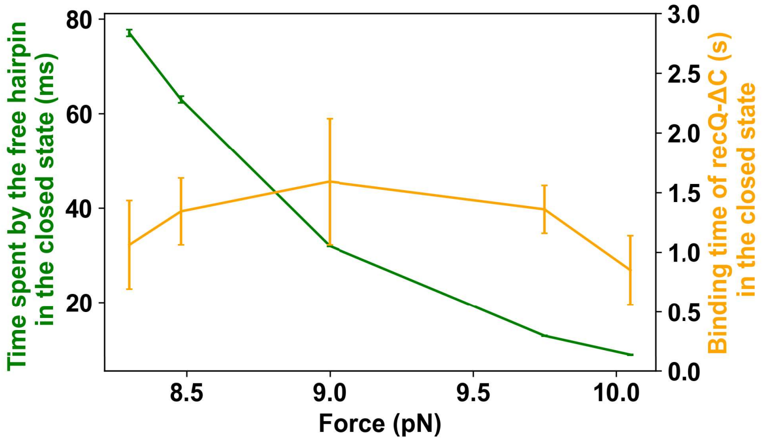

Figure S4. Force dependence of the binding of RecQ-ΔC in the closed state of the hairpin. The distribution of times spent in the closed state is fitted by a double exponential, as in figure 3. The short times (green) correspond to the times spent in the closed state by the freely fluctuating hairpin. These times are strongly dependent on the force and are used to infer the force. The long times (orange) correspond to the dissociation times of RecQ-ΔC.

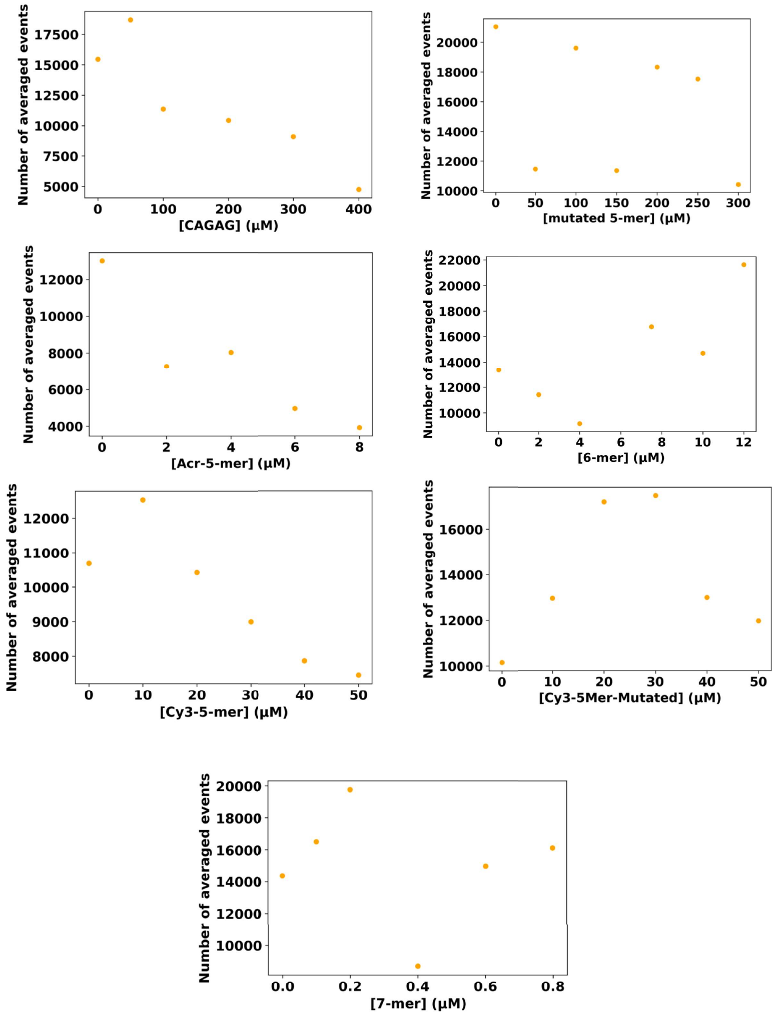

Figure S5. Number of events used to infer the parameters presented in this study.

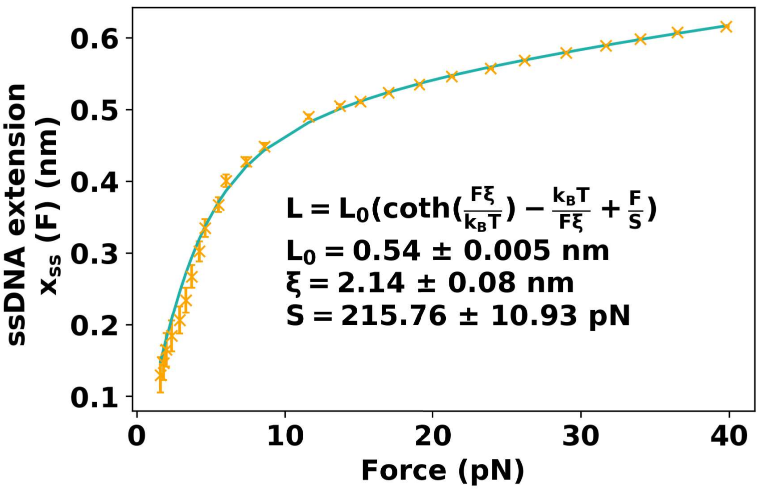

Figure S6. Experimental force-extension curve of a single-stranded DNA molecule and corresponding fit to the freely jointed chain model with extension (EFJC). Data from [35].

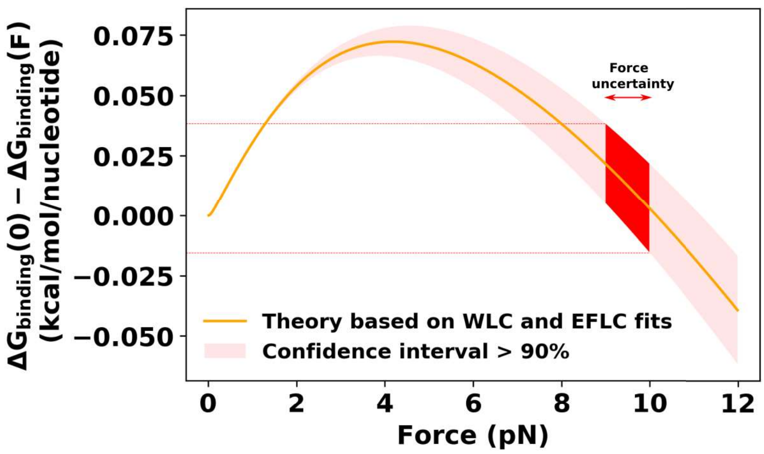

Figure S7. Contribution of the force to $\Delta G_{\text{binding}}$ computed using the worm-like chain model for double-stranded DNA (persistence length of 50 nm and maximum length per nucleotide of 0.34 nm), and the freely jointed chain model with extension for single-stranded DNA.

Oligomer	GCCAGAG	5’-MeO-Cl-Acr CAGAG
$k_{\mathrm{on}}(F)$ (μM^{-1}s^{-1})	6.90 ± 0.2	25 ± 5.7
$k_{\mathrm{off}}(F)$ (s^{-1})	4.35 ± 0.14	120 ± 34

Table S1. Binding (k_{on}) and dissociation rates (k_{off}) measured from *kinetic locking* at T = 25°C and F = 9.5 pN. Corresponding distributions from which they are inferred are shown in Figure S1

.

DNA name<br>(cf. Material and methods)	Sequence
Oli1	5’ ATTCGAAGAGCACCAGAAAGACCAAAAGACACGGTGAAGGATTAGACAGAAGAAGAC 3’-DBCO
Oli2	5’ Double-biotin TGGGAGTAGCGGATCATGATGGATGTTGCCAGCTGGTATGGAAGCTAATAGCGCCGGT 3’
HP 1 : Probe for oligo hybridization<br>When closed, has a 14 ssPAD on the leading strand and a 7 ssPAD on the lagging strand	5’ GTCTTCTTCTGTCTAATCCTTCACCGTGTCTTTTGGTCTTTCTGGTGCTCTTCGAATTTTTTTTACTGCCAGAGTTT<br>TCTCTGGCAGTGCGTGCTCGCAGTGACCGGCGCTATTAGCTTCCATACCAGCTGGCAACATCCATCATGATCCGCTACTCCCA 3’
HP 2 : Probe without ssPAD when closed	5’ GTCTTCTTCTGTCTAATCCTTCACCGTGTCTTTTGGTCTTTCTGGTGCTCTTCGAATACTGCCAGAGTTT<br>TCTCTGGCAGTACCGGCGCTATTAGCTTCCATACCAGCTGGCAACATCCATCATGATCCGCTACTCCCA 3’
HP 3 : Probe with a 14 ssPAD on the leading strand when closed	5’ GTCTTCTTCTGTCTAATCCTTCACCGTGTCTTTTGGTCTTTCTGGTGCTCTTCGAATACTGCCAGAGTTT<br>TCTCTGGCAGTGCGTGCTCGCAGTGACCGGCGCTATTAGCTTCCATACCAGCTGGCAACATCCATCATGATCCGCTACTCCCA 3’

Table S2. DNA sequences used in this paper. Names are defined in the section Material and Methods.

Supplementary Note S1 Resolution of the model supporting *kinetic locking*

We compute the exact time distribution expected for a model of *kinetic locking* that follows the scheme described in Figure 1.a.

Calling $P_f(t)$ the probability for the hairpin to be in the open free state (without oligonucleotide), $P_l(t)$ the probability to be in the open locked state (oligonucleotide hybridized), and $P_c(t)$ the probability to be in the closed state, the systems follows the dynamics :

$$\begin{bmatrix} \frac{dP_f}{dt} \\ \frac{dP_l}{dt} \\ \frac{dP_c}{dt} \end{bmatrix} = \begin{bmatrix} -(k_f+k_{\text{on}}) & k_{\text{off}} & k_u \\ k_{\text{on}} & -k_{\text{off}} & 0 \\ k_f & 0 & -k_u \end{bmatrix} \begin{bmatrix} P_f \\ P_l \\ P_c \end{bmatrix}.$$

k_f, k_u designate respectively the folding and unfolding rates of the free fluctuating probe. $k_{\text{on}}, k_{\text{off}}$ designate respectively the association and dissociation rates. The free (f) and locked (l) open states are indistinguishable. The probability to be in one of these open states is called $P_o(t) = P_f(t) + P_l(t)$. Given a hairpin that just moved to the open free state $(P_f(0) = 1, P_l(0) = 0, P_c(0) = 0)$,we are interested in $\rho_o(T)$, the distribution of times spent in the open states before a closing event $(l \to c)$ happens. Such a closing event being observable, we are not interested in the subsequent dynamics of the hairpin. Thus, the dynamics of interest is limited to the system

$$\begin{bmatrix} \frac{dP_f}{dt} \\ \frac{dP_l}{dt} \end{bmatrix} = \underbrace{\begin{bmatrix} -(k_f+k_{\text{on}}) & k_{\text{off}} \\ k_{\text{on}} & -k_{\text{off}} \end{bmatrix}}_{A} \underbrace{\begin{bmatrix} P_f \\ P_l \end{bmatrix}}_{P},$$

with $\rho_o(T) = \frac{dP_f}{dt} + \frac{dP_l}{dt} = -k_f P_f$

The matrix A has two negative eigenvalues λ_{+-}that verify :

$$\lambda_{+-} = \frac{-1}{2}\left[(k_f+k_{\text{on}}+k_{\text{off}}) \pm \sqrt{(k_f+k_{\text{on}}+k_{\text{off}})^2 - 4k_{\text{off}}k_f}\right].$$

Two corresponding eigenvectors are :

$$V_\pm = \begin{bmatrix} \alpha_\pm \\ 1 \end{bmatrix},$$

with

$$\alpha_\pm = \frac{\lambda_\pm + k_{\text{off}}}{k_{\text{on}}}.$$

We deduce the two combinations of $P_f$and P_l whose decay is exponential :

$$\alpha_\pm P_f + P_l = C_\pm e^{\lambda_\pm t}.$$

The integration constants $\alpha_\pm$ can be deduced from the initial conditions $(P_f(0) = 1, P_l(0) = 0)$, resulting in :

$$C_\pm = \alpha_\pm.$$

We deduce the expression for $P_f(t)$:

$$P_f(t) = \frac{\alpha_+ e^{\lambda_+ t} - \alpha_- e^{\lambda_- t}}{\alpha_+ - \alpha_-}.$$

And the expression for the distribution of times spent in the open states :

$$\rho(T) = k_f P_f(t) = k_f \frac{\alpha_+ e^{\lambda_+ t} - \alpha_- e^{\lambda_- t}}{\alpha_+ - \alpha_-}.$$

The mean time spent in the open states $\overline{\mathrm{T_{open}}}$ reads :

$$\begin{aligned}
\overline{\mathrm{T_{open}}} = \int_0^{+\infty} T\rho(T)dT = \frac{k_f}{\alpha_+ - \alpha_-}(\frac{\alpha_+}{{\lambda_+}^2} - \frac{\alpha_-}{{\lambda_-}^2})\\
=& \frac{k_f}{\lambda_+ - \lambda_-}(\frac{\lambda_+ + k_{\mathrm{off}}}{{\lambda_+}^2} - \frac{\lambda_- + k_{\mathrm{off}}}{{\lambda_-}^2})\\
=& k_f \frac{(\lambda_+ + k_{\mathrm{off}})\lambda_-^2 - (\lambda_- + k_{\mathrm{off}})\lambda_+^2}{{\lambda_+}^2{\lambda_-}^2(\lambda_+ - \lambda_-)}\\
=& \frac{k_f k_{\mathrm{off}}({\lambda_-}^2 - {\lambda_+}^2 + k_f \lambda_+ \lambda_-(\lambda_- - \lambda_+)}{{\lambda_+}^2{\lambda_-}^2(\lambda_+ - \lambda_-)}\\
=& \frac{-(\lambda_- + \lambda_+) + k_f}{\lambda_+ \lambda_-}\\
=& \frac{k_{\mathrm{on}} + k_{\mathrm{off}}}{k_f k_{\mathrm{off}}}.
\end{aligned}$$

, where we used that $\lambda_+ + \lambda_- = -(k_{\mathrm{on}} + k_{\mathrm{off}})$, as can be deduced from the shape of the matrix A. and $\lambda_+\lambda_- = k_f k_{\mathrm{off}}$

k_{on} is proportional to the concentration of oligonucleotides : $k_{\mathrm{on}} = k^0_{\mathrm{on}}C$. Considering that the energy of hybridization verifies : $\Delta G^0 = kT\ln(\frac{k^0_{\mathrm{on}}}{k_{\mathrm{off}}})$, we get:

$$\overline{\mathrm{T_{open}}} = \frac{1}{k_f}(1 + \frac{C}{c^0} e^{\frac{\Delta G^0}{kT}}).$$

The standard concentration c^0 is equal to 1 M so as to retrieve the standard definition of ΔG^0, which is the Gibbs energy of binding at 1 M.

Fitting $\overline{\mathrm{T_{open}}} = aC + b$ as a linear function of the concentration thus allows us to retrieve $\Delta G^0 = kT\ln(\frac{a}{b})$

Resolution in the limiting case 1: $k_{\mathrm{off}} \ll k_f$ ***and*** $k_{\mathrm{on}} \ll k_f$

This is the case where the kinetics of binding/unbinding is much slower than the kinetics of the fluctuations of the hairpin. This is the case for the binding of the 7nt-oligo and for the case of RecQ binding.

Developing at the order 2 in $\frac{k_{\mathrm{off}}}{k_f}$ and $\frac{k_{\mathrm{on}}}{k_f}$, this gives

$$\lambda_\pm = -\frac{k_f}{2}(1 + \frac{k_{\mathrm{on}} + k_{\mathrm{off}}}{k_f} \pm (1 + \frac{k_{\mathrm{on}} - k_{\mathrm{off}}}{k_f} + 2\frac{k_{\mathrm{off}}k_{\mathrm{on}}}{{k_f}^2})$$

The first order is sufficient to deduce the limit of $\lambda_\pm$. We find:

$$\begin{aligned}
\lambda_+ &= -k_f(1 + O(\tfrac{k_{\mathrm{off}} + k_{\mathrm{on}}}{k_f}))\\
\lambda_- &= -k_{\mathrm{off}}(1 + O(\tfrac{k_{\mathrm{off}} + k_{\mathrm{on}}}{k_f}))
\end{aligned}$$

In this limit, the distribution of times is thus a double exponential with very separated times that can be resolved. The long times correspond to $\frac{1}{k_{\mathrm{off}}}$ and the short times to $\frac{1}{k_f}$.

On the order hand, the proportion of the short times is:

$$
\begin{aligned}
\alpha = & \frac{k_f \alpha_+}{-\lambda_+(\alpha_+ - \alpha_-)} \\
= & \frac{k_f(\lambda_+ + k_{\text{off}})}{\lambda_+(\lambda_+ - \lambda_-)} \\
= & \frac{-k_f{}^2(1 + \frac{k_{\text{on}}}{k_f} - \frac{k_{\text{off}}}{k_f})}{-k_f(1 + \frac{k_{\text{on}}}{k_f})(k_f + k_{\text{on}} - k_{\text{off}})} \\
= & \frac{(1 + \frac{k_{\text{on}}}{k_f} - \frac{k_{\text{off}}}{k_f})}{(1 + \frac{k_{\text{on}}}{k_f})(1 + \frac{k_{\text{on}}}{k_f} - \frac{k_{\text{off}}}{k_f})} \\
= & 1 - \frac{k_{\text{on}}}{k_f}
\end{aligned}
$$

Resolution in the limiting case 2: $k_{\text{off}} \gg k_f$ ***and*** $k_{\text{on}} \gg k_f$

This is the case where the kinetics of binding/unbinding is much faster than the kinetics of the fluctuations of the hairpin. This is the case for the binding of short oligonucleotides (5,6 bases).

In this case, the first eigenvalue reads:

$$
\lambda_+ = (k_{\text{off}} + k_{\text{on}}) \tag{2}
$$

This corresponds to short times that cannot be observed.

The second value reads, at the first order in $\frac{k_f}{k_{\text{on}}+k_{\text{off}}}$:

$$
\lambda_- = -\frac{1}{2}\left[(k_f + k_{\text{on}} + k_{\text{off}}) - (k_{\text{on}} + k_{\text{off}})(1 + \frac{k_f}{k_{\text{on}} + k_{\text{off}}} - \frac{2k_f k_{\text{off}}}{(k_{\text{on}} + k_{\text{off}})^2}\right] \tag{3}
$$

$$
= -\frac{k_{\text{off}} k_f}{k_{\text{off}} + k_{\text{on}}} \tag{4}
$$

$$
= -\frac{k_f}{1 + \frac{k_{\text{on}}}{k_{\text{off}}}} \tag{5}
$$

This eigenvalue corresponds to a time that evolves in the same way that the average time $\overline{T_{\text{open}}}$, increasing linearly with the concentration. Thus, in this regime, the observable distribution follows a simple exponential distribution that does not allow measuring k_{on} and k_{off}. Only their ratio, and thus ΔG, can be measured.

Supplementary Note S2 Effect of the force on the measure of $\Delta G_{\text{binding}}$

In this section we detail the impact of the force on the estimation of the binding free energy of oligos $\Delta G_{\text{binding}}$. This effect can be calculated if we know the precise force-extension curves of single-stranded DNA $x_{\text{ss}}(F)$ and of double-stranded DNA $x_{\text{ds}}(F)$. In this case, the variation of free energy reads :

$$
\Delta G_{\text{binding}}(F) = \Delta G_{\text{binding}}(0) + \int_0^{x_{\text{ss}}(F)} x_{\text{ss}}(f)\text{d}f - \int_0^{x_{ds}(F)} x_{ds}(f)\text{d}f \tag{6}
$$

We recall the derivation of this formula for readers that would not be familiar with it in the Supplementary Note S3.

The force-extension curve of double-stranded DNA $x_{ds}(f)$ is well-established (the worm-like chain model fits it well for forces < 15 pN). The force-extension curve of single-stranded DNA $x_{ss}(f)$ is derived from the fit of a long ssDNA by the freely jointed chain model with extension (EFJC, Figure S6). The corresponding estimation of $\Delta G_{\text{binding}}$ as a function of the force is shown on Figure S7. The confidence interval is based on the errors made on the fit of the ssDNA by the EFJC model. The figure shows that the contribution of the force to $\Delta G_{\text{binding}}$ is smaller than 0.04 kcal/mol/nucleotide in the force range of our experiments.

Supplementary Note S3 Derivation of the force dependence of $\Delta G_{\text{binding}}(F)$

First, we remind how the force-extension curves allow computing the free energy of DNA at various extensions $G_{ss/ds}(x_{ss/ds})$. When pulled at a force F, a molecule adopts an extension that minimizes the free energy of the whole system, consisting of the molecule and the magnetic bead, and that reads:

$$G_{\text{total}}(x) = G_{ss/ds}(x) - Fx. \quad (7)$$

Minimizing G_{total} with respect to x leads to :

$$\frac{\partial G_{ss/ds}}{\partial x} = F \quad (8)$$

The observed $x_{ss/ds}$ is the value of the extension x that minimizes this free energy and is the one measured experimentally. It allows to define a function $x_{ss/ds}(F)$. We can also define the reciprocal function $f(x_{ss/ds})$ that corresponds to the force F needed to observe the extension $x_{ss/ds}$. In particular, it means that we can compute $G_{ss/ds}(F)$ by integrating the observed extension curve with respect to x :

$$G_{ss/ds}(F) = G_{ss/ds}(x_{ss/ds}(F)) = G_{ss/ds}(0) + \int_{x'=0}^{x'=x_{ss/ds}(F)} f(x')\mathrm{d}x' \quad (9)$$

$\Delta G_{\text{binding}}(F)$ is the difference of free energy between a double-stranded DNA pulled at a force F (final state, hybridized) and two single-stranded DNA, the first being pulled at force F, and the other, free in solution, on which no force is applied :

$$\Delta G_{\text{binding}}(F) = \underbrace{G_{ds}(F) - Fx_{ds}(F)}_{\textbf{bead + dsDNA pulled at F}} - \underbrace{(G_{ss}(F) - Fx_{ss}(F))}_{\textbf{bead +ssDNA pulled at F}} - \underbrace{G_{ss}(0)}_{\textbf{free oligo}} \quad (10)$$

Thus, using the equation just above :

$$\Delta G_{\text{binding}}(F) = \left(G_{ds}(0) + \int_{x'=0}^{x'=x_{ds}(F)} f(x')\mathrm{d}x' - Fx_{ds}(F)\right) - \left(G_{ss}(0) + \int_{x'=0}^{x'=x_{ss}(F)} f(x')\mathrm{d}x' - Fx_{ss}(F)\right) - G_{ss}(0) \quad (11)$$

In the two parentheses, we recognize an integration by part, and thus :

$$\Delta G_{\text{binding}}(F) = \left(G_{ds}(0) + \int_{f=0}^{f=F} x_{ds}(f)\mathrm{d}f\right) - \left(G_{ss}(0) + \int_{f=0}^{f=F} x_{ss}(f)\mathrm{d}f\right) - G_{ss}(0) \quad (12)$$

$$= \Delta G_{\text{binding}}(0) + \int_{f=0}^{f=F} x_{ds}(f)\mathrm{d}f - \int_{f=0}^{f=F} x_{ss}(f)\mathrm{d}f \quad (13)$$

35. Manosas, M., Spiering, M. M., Ding, F., Croquette, V. & Benkovic, S. J. Collaborative coupling between polymerase and helicase for leading-strand synthesis. Nucleic Acids Res. 40, 6187–6198 (2012).
