# Supplementary material for: Single-molecule kinetic locking allows fluorescence-free quantification of protein/nucleic-acid binding
Source: Commun Biol. 2021 Sep 15;4:1083. doi: 10.1038/s42003-021-02606-z (PMC8443601; doi:10.1038/s42003-021-02606-z)
Supplement: Supplementary file 3 — Description of Supplementary Files [file 42003_2021_2606_MOESM3_ESM.pdf]

Description of Additional Supplementary Files

File name: Supplementary Data 1

Description: Data underlying graphs and charts.

File name: Supplementary Data 2

Description: Individual time points supporting the fits of the distributions and the inference procedures.
